# Development and assessment of novel machine learning models to predict the probability of postoperative nausea and vomiting for patient-controlled analgesia

**DOI:** 10.1038/s41598-023-33807-7

**Published:** 2023-04-20

**Authors:** Min Xie, Yan Deng, Zuofeng Wang, Yanxia He, Xingwei Wu, Meng Zhang, Yao He, Yu Liang, Tao Li

**Affiliations:** 1 Laboratory of Mitochondria and Metabolism, Department of Anesthesiology, National Clinical Research Center for Geriatrics, West China Hospital, Sichuan University, No. 37 Wainan Guoxue Road, Chengdu, 610041 Sichuan People’s Republic of China; 2 Department of Anesthesiology, Sichuan Academy of Medical Sciences & Sichuan Provincial People’s Hospital, Chengdu, 610072 Sichuan People’s Republic of China; 3 Department of Anesthesiology, Chengdu First People’s Hospital, Chengdu, Sichuan 610017 People’s Republic of China; 4 Personalized Drug Therapy Key Laboratory of Sichuan Province, Department of Pharmacy, Sichuan Academy of Medical Sciences & Sichuan Provincial People’s Hospital, Chengdu, Sichuan 610072 People’s Republic of China

**Keywords:** Risk factors, Signs and symptoms

## Abstract

Postoperative nausea and vomiting (PONV) can lead to various postoperative complications. The risk assessment model of PONV is helpful in guiding treatment and reducing the incidence of PONV, whereas the published models of PONV do not have a high accuracy rate. This study aimed to collect data from patients in Sichuan Provincial People’s Hospital to develop models for predicting PONV based on machine learning algorithms, and to evaluate the predictive performance of the models using the area under the receiver characteristic curve (AUC), accuracy, precision, recall rate, F1 value and area under the precision-recall curve (AUPRC). The AUC (0.947) of our best machine learning model was significantly higher than that of the past models. The best of these models was used for external validation on patients from Chengdu First People’s Hospital, and the AUC was 0.821. The contributions of variables were also interpreted using SHapley Additive ExPlanation (SHAP). A history of motion sickness and/or PONV, sex, weight, history of surgery, infusion volume, intraoperative urine volume, age, BMI, height, and PCA_3.0 were the top ten most important variables for the model. The machine learning models of PONV provided a good preoperative prediction of PONV for intravenous patient-controlled analgesia.

## Introduction

Postoperative nausea and vomiting (PONV) is the most common adverse event after general anesthesia, appearing in 40% of Asian and European American populations and up to 80% of high-risk cases^1,2^. The complications of PONV include surgical wound dehiscence, dehydration, rupture of the esophagus, bleeding, and increased intraocular and intracranial pressures, which lead to increased health costs, longer stays in the hospital and ultimately lower patient satisfaction^3^.

Studies have indicated that risk assessment for PONV is helpful in reducing the incidence of PONV and guiding treatment^4,5^. In recent years, various nausea and vomiting risk scores or prediction models have been proposed. To date, the simplified Apfel risk score is the most widely used prediction model and includes four variables: female sex, history of motion sickness or postoperative nausea or vomiting, smoking, and the use of postoperative opioids^2^. The Koivuranta score includes the 4 Apfel risk predictors as well as length of surgery > 60 min^6^. Chae’s team also proposed a predictive model is about for intravenous fentanyl patient-controlled analgesia^7^.

Previous studies have raised several questions. For instance, most of these models had no antiemetic drugs during the operation^2,6^, and the anesthesia modality was either inhalation anesthesia or intravenous anesthesia only^2,8^. However, some anesthesiologists may choose intravenous combined inhalation anesthesia, and give antiemetic drugs during surgery; the analgesic formula was also determined according to the specific situation of the patient and anesthesiologist's administration habits. The notion of Enhanced recovery after surgery (ERAS) has been widely accepted^9^, and anesthesiologists in our hospital routinely recommend patients to receive postoperative patient-controlled analgesia (PCA) after surgery. Therefore, the accuracy of past models in predicting nausea and vomiting was not high. It is well known that the area under the receiver characteristic curve (AUC) is used as an evaluation index to judge the quality of a model, and higher AUC values indicate better prediction accuracy of the model. However, the AUC values of most past models did not exceed 0.70^1^. Thus, it may be that in addition to the predictive factors proposed by the previous models, there are other important factors contributing to the occurrence of nausea and vomiting in patients that have not been identified.

In recent years, with the rise of artificial intelligence, machine learning algorithms have been increasingly applied to develop predictive models^10^. Shim et al. used machine learning algorithms to develop predictive models of PONV, but the AUC (0.686) was not high^11^. The difference in AUC values may be related to the data preprocessing methods and the machine learning algorithms^12^.

The purpose of this study was to create prediction models of PONV by collecting clinical data from adult patients receiving PCA based on machine learning algorithms, and to determine the key factors affecting PONV.

## Results

### Patient characteristics

A total of 2222 patients of Sichuan Provincial People’s Hospital were enrolled in the survey, including 1777 patients in the training set and 445 patients in the test set. An average of 13.91% (309/2222) of patients suffered from PONV. The characteristics of different variables of patients from Sichuan Provincial People’s Hospital during the perioperative period can be found in Supplementary Table S1. A total of 20.52% (110/536) of patients in Chengdu First People’s Hospital suffered from PONV.

### Dataset building

We selected 25 perioperative variables for model construction. After data preprocessing, 21 variables (type of surgery, age, sex, height, weight, BMI, past medical history, history of surgery, laparoscopic surgery, operative duration, infusion volume, intraoperative urine volume, blood loss, antiemetics used during surgery, nonopioids used during surgery, remifentanil consumption, sufentanil consumption, ephedrine, dexmedetomidine, PCA regimen, history of motion sickness and/or PONV) were retained, and 6 variables (ASA, smoking, drinking, midazolam, propofol and volatile anesthetics) were deleted. More than 85% of patients at the Sichuan Provincial People’s Hospital had an ASA class II, were nonsmokers, nondrinkers, and used midazolam, propofol, and inhaled anesthetics in surgery, so the six variables were not included in the model because the coefficients of variation were too small.

### Model establishment

A total of 54 prediction models were established by nine machine learning algorithms, three variable selection methods, two data sampling methods, and random forest imputing methods. Samples from the test set were used to evaluate the impact of different data processing methods or machine learning algorithms on model predictive performance. The results showed that differences in model predictive performance exist by different data filling, data sampling, variable selection and machine learning algorithms (Tables 1, 2).

**Table 1:** The effect of different machine learning algorithms on model prediction performance using BSMOTE sampling method.

Inputing method	Sampling method	Selection methods	Model name	AUC	Accuracy	Precision	Recall	F1 Score	Specificity
RF	BSMOTE	BOR	XGB	0.6381	0.6049	0.5751	0.8031	0.6702	0.4067
RF	BSMOTE	BOR	RF	0.6382	0.6049	0.5751	0.8031	0.6702	0.4067
RF	BSMOTE	BOR	SVC	0.6379	0.6049	0.5751	0.8031	0.6702	0.4067
RF	BSMOTE	BOR	KNN	0.5206	0.4974	0.4285	0.0155	0.0300	0.9792
RF	BSMOTE	BOR	CB	0.6374	0.6062	0.5762	0.8031	0.6709	0.4093
RF	BSMOTE	BOR	LR	0.6459	0.6113	0.5820	0.7901	0.6703	0.4326
RF	BSMOTE	BOR	MLP	0.5000	0.5000	0.5000	1.0000	0.6666	0
RF	BSMOTE	BOR	SGD	0.6501	0.6101	0.5812	0.7875	0.6688	0.4326
RF	BSMOTE	BOR	DT	0.6370	0.6062	0.5762	0.8031	0.6709	0.4093
RF	BSMOTE	LA	XGB	0.9443	0.8795	0.9126	0.8393	0.8744	0.9196
RF	BSMOTE	LA	RF	0.9348	0.8471	0.8941	0.7875	0.8374	0.9067
RF	BSMOTE	LA	SVC	0.8809	0.7992	0.8290	0.7538	0.7896	0.8445
RF	BSMOTE	LA	KNN	0.7753	0.6981	0.6703	0.7797	0.7209	0.6165
RF	BSMOTE	LA	CB	0.9469	0.8575	0.8942	0.8108	0.8505	0.9041
RF	BSMOTE	LA	LR	0.9106	0.8341	0.7972	0.8963	0.8439	0.7720
RF	BSMOTE	LA	MLP	0.8929	0.7914	0.8482	0.7098	0.7729	0.8730
RF	BSMOTE	LA	SGD	0.9350	0.8484	0.8120	0.9067	0.8567	0.7901
RF	BSMOTE	LA	DT	0.8606	0.8290	0.8223	0.8393	0.8307	0.8186
RF	BSMOTE	RFE	XGB	0.9396	0.8691	0.8589	0.8834	0.8710	0.8549
RF	BSMOTE	RFE	RF	0.9354	0.8523	0.8469	0.8601	0.8534	0.8445
RF	BSMOTE	RFE	SVC	0.9089	0.8536	0.8403	0.8730	0.8564	0.8341
RF	BSMOTE	RFE	KNN	0.9249	0.7992	0.9601	0.6243	0.7566	0.9740
RF	BSMOTE	RFE	CB	0.9387	0.8626	0.8414	0.8937	0.8668	0.8316
RF	BSMOTE	RFE	LR	0.9072	0.8316	0.8062	0.8730	0.8383	0.7901
RF	BSMOTE	RFE	MLP	0.9367	0.8626	0.8500	0.8808	0.8651	0.8445
RF	BSMOTE	RFE	SGD	0.9277	0.8419	0.8235	0.8704	0.8463	0.8134
RF	BSMOTE	RFE	DT	0.9201	0.8601	0.8582	0.8626	0.8604	0.8575

*BSMOTE* borderline synthetic minority oversampling technique, *BOR* boruta screening, *RFE* recursive feature elimination, *LA* lasso screening, *XGB* extreme gradient boosting, *SVC* support vector classify, *KNN* K-nearest neighbor, *CB* category boosting, *LR* logistic regression, *MLP* multilayer perceptron, *SGD* stochastic gradient descent, *DT* decision tree, *AUC* area under the curve.

**Table 2:** The effect of different machine learning algorithms on model prediction performance using SMOTE sampling method.

Inputing method	Sampling method	Selection methods	Model name	AUC	Accuracy	Precision	Recall	F1 Score	Specificity
RF	SMOTE	BOR	XGB	0.5286	0.5092	0.2751	0.4765	0.3488	0.5217
RF	SMOTE	BOR	RF	0.5296	0.5092	0.2751	0.4765	0.3488	0.5217
RF	SMOTE	BOR	SVC	0.5351	0.5074	0.2741	0.4765	0.3480	0.5191
RF	SMOTE	BOR	KNN	0.4893	0.7203	0.3333	0.0134	0.0258	0.9897
RF	SMOTE	BOR	CB	0.5301	0.5092	0.2751	0.4765	0.3488	0.5217
RF	SMOTE	BOR	LR	0.5149	0.4722	0.2763	0.5637	0.3708	0.4373
RF	SMOTE	BOR	MLP	0.5995	0.2759	0.2759	1	0.4325	0
RF	SMOTE	BOR	SGD	0.5153	0.4351	0.3020	0.7986	0.4383	0.2966
RF	SMOTE	BOR	DT	0.5242	0.5037	0.2551	0.4161	0.3163	0.5370
RF	SMOTE	LA	XGB	0.9055	0.8444	0.7685	0.6241	0.6888	0.9283
RF	SMOTE	LA	RF	0.9010	0.8129	0.7448	0.4899	0.5910	0.9360
RF	SMOTE	LA	SVC	0.8926	0.8314	0.7042	0.6711	0.6872	0.8925
RF	SMOTE	LA	KNN	0.7402	0.7074	0.4771	0.630872	0.5433	0.7365
RF	SMOTE	LA	CB	0.9124	0.8425	0.7461	0.651007	0.6953	0.9156
RF	SMOTE	LA	LR	0.8811	0.8074	0.6203	0.778523	0.6904	0.8184
RF	SMOTE	LA	MLP	0.8439	0.8092	0.6796	0.583893	0.6281	0.8951
RF	SMOTE	LA	SGD	0.9013	0.8259	0.6519	0.791946	0.7151	0.8388
RF	SMOTE	LA	DT	0.8556	0.7925	0.6069	0.704698	0.6521	0.8260
RF	SMOTE	RFE	XGB	0.9097	0.8537	0.7243	0.758389	0.7409	0.8900
RF	SMOTE	RFE	RF	0.9097	0.8592	0.7417	0.751678	0.7466	0.9002
RF	SMOTE	RFE	SVC	0.8967	0.8425	0.6860	0.791946	0.7352	0.8618
RF	SMOTE	RFE	KNN	0.8758	0.8277	0.7692	0.536913	0.6324	0.9386
RF	SMOTE	RFE	CB	0.9170	0.8388	0.6684	0.825503	0.7387	0.8439
RF	SMOTE	RFE	LR	0.8807	0.8055	0.6182	0.771812	0.6865	0.8184
RF	SMOTE	RFE	MLP	0.9068	0.8407	0.6800	0.798658	0.7345	0.8567
RF	SMOTE	RFE	SGD	0.9012	0.8240	0.6377	0.838926	0.7246	0.8184
RF	SMOTE	RFE	DT	0.8812	0.8629	0.7516	0.751678	0.7516	0.9053

*SMOTE* synthetic minority oversampling technique, *BOR* boruta screening, *RFE* recursive feature elimination, *LA* lasso screening, *XGB* extreme gradient boosting, *SVC* support vector classify, *KNN* K-nearest neighbor, *CB* category boosting, *LR* logistic regression, *MLP* multilayer perceptron, *SGD* stochastic gradient descent, *DT* decision tree, *AUC* area under the curve.

### Model evaluation

**Figure 1:**
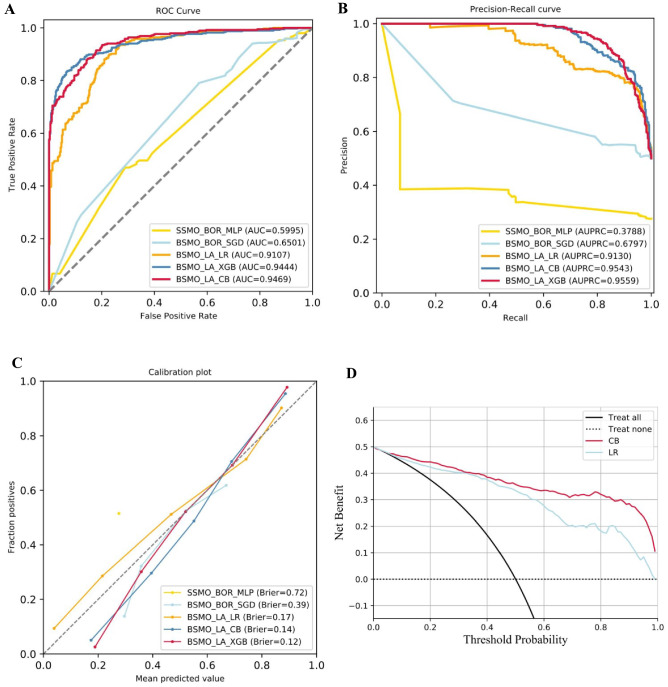
The performance of the five best models. (**A**) The ROC curves of the five best models. (**B**) The precision-recall curves of the five best models. (**C**) The calibration curves of the five best models. (**D**) Decision curve analysis of the models. *SMOTE* synthetic minority oversampling technique, *BOR* boruta screening, *MLP* multiple-layer perceptron, *BSMOTE* borderline synthetic minority oversampling technique, *SGD* stochastic gradient descent, *LA* lasso screening, *LR* logistic regression, *XGB* extreme gradient boosting, *CB* category boosting, *AUC* area under the curve, *AUPRC* area under the precision-recall curve.

The AUC, accuracy, precision, recall rate, F1 value, and AUPRC were used to evaluate the performance of the models. The AUCs of the five best models were 0.5995, 0.6501, 0.9107, 0.9444, and 0.9469. The model using the Lasso screening method, BSMOTE and CatBoost algorithms had the best performance (AUC = 0.9469). We used the best model in patients from Chengdu First People’s Hospital for external validation, and the AUC also reached 0.8211 (Table 3). To determine the clinical usefulness of the model by quantifying the net benefits, decision curve analyses for this prediction model were performed. The characteristic curve of the five best models is shown in Fig. 1.

**Table 3:** Predictive performance indicators of the five best models and Chengdu First People’s Hospital.

Model	AUC	Accuracy	Precision	Recall	F1 Score	Specificity
SMOTE_BOR_MLP	0.5995	0.2759	0.2759	1	0.4325	0
BSMOTE_BOR_SGD	0.6501	0.6101	0.5813	0.7876	0.6689	0.4326
BSMOTE_LA_LR	0.9107	0.8342	0.7972	0.8964	0.8439	0.7720
BSMOTE_LA_XGB	0.9444	0.8795	0.9127	0.8394	0.8745	0.9197
BSMOTE_LA_CB	0.9469	0.8575	0.8943	0.8108	0.8505	0.9041
FIRST _HOSPITAL	0.8211	0.7230	0.4098	0.7618	0.5325	0.7128

*SMOTE* synthetic minority oversampling technique, *BOR* boruta screening, *MLP* multilayer perceptron, *BSMOTE* borderline synthetic minority oversampling technique, *SGD* stochastic gradient descent, *LA* lasso screening, LR logistic regression, *XGB* extreme gradient boosting, *CB* category boosting, *AUC* area under the curve.

### Model interpretation

**Figure 2:**
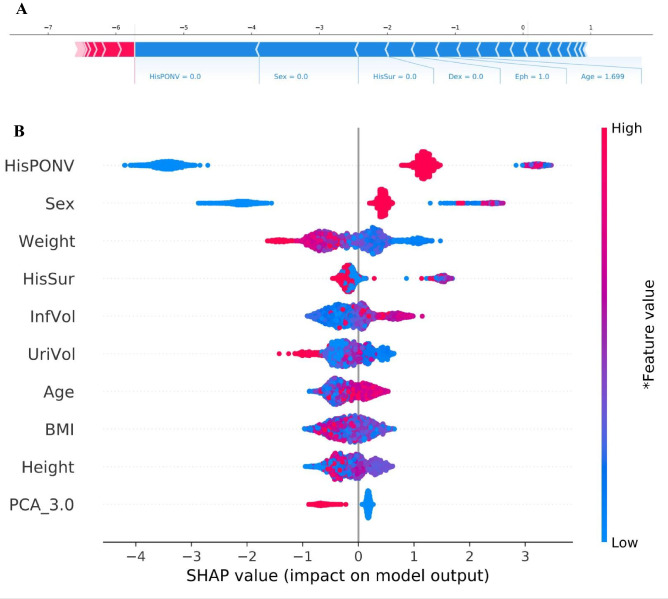
Variable contribution to models by SHAP Value. (**A**) Contribution of each feature value in one sample. (**B**) The SHAP value of the top 10 combination variables. The higher the SHAP value of a variable is, the more likely nausea and vomiting. The redder the color of the variable, the larger the value, and the bluer the color of the variable are, the smaller the value. *SHAP* SHapley Additive ExPlanation, *History of motion sickness and/or PONV* HisPONV, *history of surgery* HisSur, *InfVol* Infusion volume, *UriVol* Intraoperative urine volume, *BMI* Body Mass Index, *PCA_3.0* PCA regimen 3 (sufentanil, nonsteroidal anti-inflammatory drugs in the analgesic pump).

We used the SHapley Additive ExPlanation (SHAP) value to explain the contribution of the variables to the model. SHAP estimated the contribution of each feature value in each sample to the prediction in Fig. 2A. No history of motion sickness and/or PONV, male, no history of surgery, no dexmedetomidine use, no ephedrine use and young age provided a negative contribution. The number of possible combinations of variables increased exponentially as the number of variables increased, and the selection order of combined variables affected the SHAP value. The SHAP value of the top 10 combination variables is shown in Fig. 2B. The results showed that history of motion sickness and/or PONV, sex, weight, history of surgery, infusion volume, intraoperative urine volume, age, BMI, height, and PCA_3.0 were the top ten most important variables for the model. In our study, the analgesic pump formulation had three options: the first formulation was sufentanil, the second formulation was hydromorphone, and the third formulation was sufentanil and nonsteroidal anti-inflammatory drugs (NSAIDs). PCA_3.0 is the third option for the formulation of analgesic pumps.

For the prediction model, the higher the SHAP value of a variable was the more likely PONV. The redder the color of the variable were the larger the value, and the bluer the color of the variable were the smaller the value. Females, older patients, short patients, patients with a history of motion sickness and/or PONV, light weight individuals with heavy infusions, low intraoperative urine volume, and small BMI, and patients who have not had surgery and have not chosen a third analgesic pump formulation were all more likely to experience PONV.

### Sample size assessment

**Figure 3:**
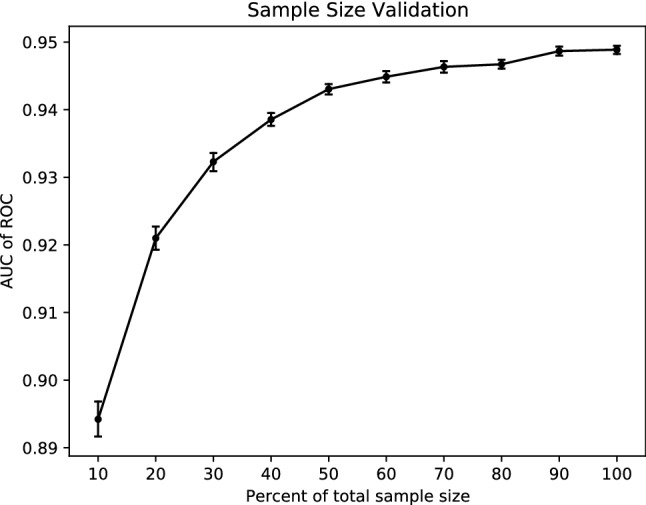
The impact of sample data size on model performances. *AUC* area under the curve, *ROC* operator characteristic.

With the continuously increasing size of the sample data, the AUC values of the testing sets continued to increase, which shows a sufficient sample size was included in this study (Fig. 3).

## Discussion

In our research, we developed a total of 54 models for the prediction of PONV in patients with PCA at Sichuan Provincial People’s Hospital. The AUC of five best models was 0.9469. Meanwhile, the best model was validated on patients from Chengdu First People’s Hospital, and the AUC value also reached 0.8211.

Apfel and his colleagues used six models, Apfel, Koivuranta, Sinclair, Palazzo, Gan and Scholz, to study PONV in the European population undergoing orthopedic surgery, gastrointestinal surgery, otorhinolaryngology surgery, and gynecological surgery, and the AUCs of these scoring systems were 0.61 to 0.68^13^. Jorg M. Engel’s team used the Koivuranta, simplified Apfel, Sinclair, and Junger risk scoring systems to predict PONV in otolaryngology surgery, and the AUC of the Sinclair and Junger models was 0.70^14^. Wu et al. studied five popular scoring systems, Apfel, Koivuranta, Palazzo and Evans, Simplified Apfel and Simplified Koivuranta risk score systems, which were validated in a Taiwanese population; the AUCs of these scoring systems ranged from 0.62 to 0.67^1^.The AUC of Shim’s machine learning models ranged from 0.561 to 0.686^11^. Our models’ AUC (0.9469) was significantly higher than that of other models, which may be because our model was constructed using the BSMOTE sampling method, the Lasso screening method, and the CatBoost machine learning method. The different data preprocessing methods and different machine learning algorithms can lead to different prediction performances of the model^12^.

Our study collected almost all of the variables in the perioperative period, probably the largest number of variables in the current prediction model. In prior studies that developed predictive models for PONV, a part of the studies explicitly stated that the trials were conducted without the use of antiemetic drugs, and another part of the studies did not specify whether the patients used antiemetic drugs. All studies did not include antiemetic drugs as a predictive model variable, and therefore our study did not include them as a predictive study variable either. A history of surgery, intraoperative urine volume, and blood loss were included as variables in our study. Past models have only presented variables that affect PONV and have not assessed the contribution of each variable to the effect of nausea and vomiting occurrence. In contrast, our model explained the contribution of the different variables on the occurrence of PONV by SHAP. A history of motion sickness and/or PONV, sex, and weight were the three most influential variables among all variables. Weight, history of surgery, intraoperative urine volume and height were used for the first time as factors to predict PONV. However, the reasons for the occurrence of PONV due to these variables are not clear and need to be further investigated.

Two variables, history of motion sickness and/or PONV, and sex, also appear in previous predictive models^2,4^ and are recognized as important variables influencing PONV. The cause of nausea and vomiting due to a history of motion sickness and/or PONV is not particularly clear and may be related to genetics. Previous studies have suggested that the link between genetics and PONV may be the result of anesthetic agent administration and surgical factors, interacting with various small genomic differences between individuals^5^. The use of NSAIDs instead of opioids for analgesia is known to reduce the occurrence of PONV, and our model supports this finding^15^. Weight, height and BMI were also considered as factors influencing PONV in our model. Patients with lighter body weight, shorter height and smaller BMI were more likely to experience PONV. In Johansson's study, the included patients had a BMI of 28.3 ± 6.9, and patients with BMI > 35 kg/m^2^ were more likely to experience PONV^16^, whereas the patients in our study had a BMI of 23.51 ± 3.54, and only 20 patients had a BMI > 35 kg/m^2^. Differences between races may be responsible for the different results.

In our prediction model, a higher intraoperative infusion volume and lower intraoperative urine volume led to an increased probability of PONV. During surgery, when a patient is infused with a high volume of fluid while intraoperative urine volume remains low, there may be a reduction in the effective circulating intravascular volume and an inadequate amount of fluid infusion, or there is a condition of renal impairment^17^. Jewer found that adequate perioperative intravenous crystalloid infusion administration reduces PONV in ASA I to II patients receiving general anaesthesia for short surgical procedures^18^. In other words, inadequate infusion may lead to an increased incidence of PONV. This finding is in line with our conclusion.

Our prediction model also showed that older age may lead to a greater risk of PONV in patients. However, the Apfel team's prediction model for PONV in outpatients suggests that patients under the age of 50 are more likely to experience PONV^18^. In our study, segmentation of patient age was not performed, but rather patient age was included in the machine learning model as a continuous variable for the study. The reason for the difference may be related to the sample size, the number of variables, and the differences in ethnicity, so further studies with larger samples are needed.

Although multiple comprehensive guidelines and risk assessment models have been published on the subject, PONV continues to plague the surgical population. The most likely reason is the lack of compliance with nausea and vomiting prevention guidelines^19^. Pysyk et al. reported that the incidence of PONV was reduced by annual anesthesiologist performance feedback urging the use of antiemetic medications^20^. Rajan et al. concluded that identifying high-risk patients through the use of predictive models for PONV, taking intraoperative and postoperative combinations of multiple types of antiemetic medications, or changing anesthesia can further reduce the incidence of PONV^19^. Therefore, it is possible to reduce the incidence of PONV by using assessment tools to proactively guide clinical practice.

### Limitations

This study has some limitations. First, the study population came from only two medical institutions in the same city in western China and did not include patients who underwent head and neck surgery (this group of patients did not undergo routine postoperative intravenous analgesia). The sample may be underrepresentative, and there may be sample selection bias, which may have some impact on the extrapolation of the results of this study. Second, inhaled anesthetics and propofol were used in most patients, and it was not possible to determine the effect of these two drugs separately on PONV.

## Conclusions

In conclusion, we not only constructed machine learning models for predicting PONV, but also identified factors affecting the occurrence of PONV. The three indicators of history of motion sickness and/or PONV, female sex, and light weight are especially important for anesthesiologists and surgeons to take consider. We hope our prediction model can serve as a reference for clinical decision-making.

## Methods

### Data sources

Patients who received surgical procedures in Sichuan Provincial People’s Hospital from October 2021 to March 2022 were included in this study and were used for the modeling. To externally validate the predictive model, we retrospectively collected data related to patients who underwent surgical procedures from February to July 2022 in Chengdu First People’s Hospital. The inclusion criteria were as follows: patients (aged ≥ 18 years) who underwent general anesthesia and postoperative PCA. Exclusion criteria: patients admitted to the intensive care unit (ICU) after surgery. The Ethics Committee of Sichuan Provincial People’s Hospital (approval no. 2022-49-1) and Chengdu First People’s Hospital (approval no.2022-HXKT-011) approved this retrospective analysis of routinely collected data and waived patient consent. This study was registered at the Chinese Clinical Trial Registry (Registration number ChiCTR2200056097, principal investigator: Min Xie, http://www.chictr.org.cn/showproj.aspx?proj=151192, date of registration: February 1, 2022). Our study methods were performed in accordance with the guidelines and regulations of the clinical registry. All private personal information was protected and removed during the process of analysis and publication.

### Data collection and outcome definition

Recent studies have found that some factors, including the type of surgery^14,21^, anesthesia drugs^22–25^, age^18,26^, perioperative fasting^27^, infusion volume^28^, anxiety^29^, inhalation anesthetics^27^, body mass index (BMI)^16^ and operative duration^6^ are related to the PONV. Therefore, we included as many variables as possible in our prediction model. Some of these variables were not present in past studies, such as the history of surgery, intraoperative urine volume and blood loss.

The clinical information of patients was retrospectively collected by the Hospital Information System (HIS) and scientific research assistants. The medical history and condition of patients were collected by surgeons and recorded in the HIS. The anesthetic protocol and postoperative analgesia formula were determined by the patient’s anesthesiologist and were not standardized. The nurses recorded the occurrence of PONV and rescue analgesics in the PACU. When the patient returned to the ward, the anesthesia nurse followed up with PONV and other side effects for 24 to 72 h after the procedure. The anesthesia nurse asked the patients questions about vomiting and nausea, such as, “Have you vomited or had dry-retching?”, “Have you experienced a feeling of nausea?” and “When did you experience PONV?”. PONV was considered to have occurred when patients had nausea, vomiting, or both. At the same time, the patient’s resting pain score and movement pain score were measured by a visual analog scale (VAS). Most PONV occurs within 24 h after surgery and decreases in degree and incidence with time^30^. In this study, only PONV and movement pain scores that occurred within 24 h postoperatively were recorded. All data were collected from the HIS by scientific research assistants, who were blinded to the study hypothesis.

### Data preprocessing

Variables with missing data > 90%, a single category > 90%, a coefficient of variation < 0.01 were deleted^31^.

### Data partitioning and dataset building

The patients at Sichuan Provincial People’s Hospital were divided into a training set and test set at a ratio of 8:2 and were used to train and test models respectively. Patients at Chengdu First People’s Hospital were used to detect the developed models externally.

Some of the missing clinical information, such as height, weight, history of motion sickness, and/or PONV, was collected by the research assistant on the phone; the other missing data, such as PCA regimen, were filled in using the random forest method.

**Figure 4:**
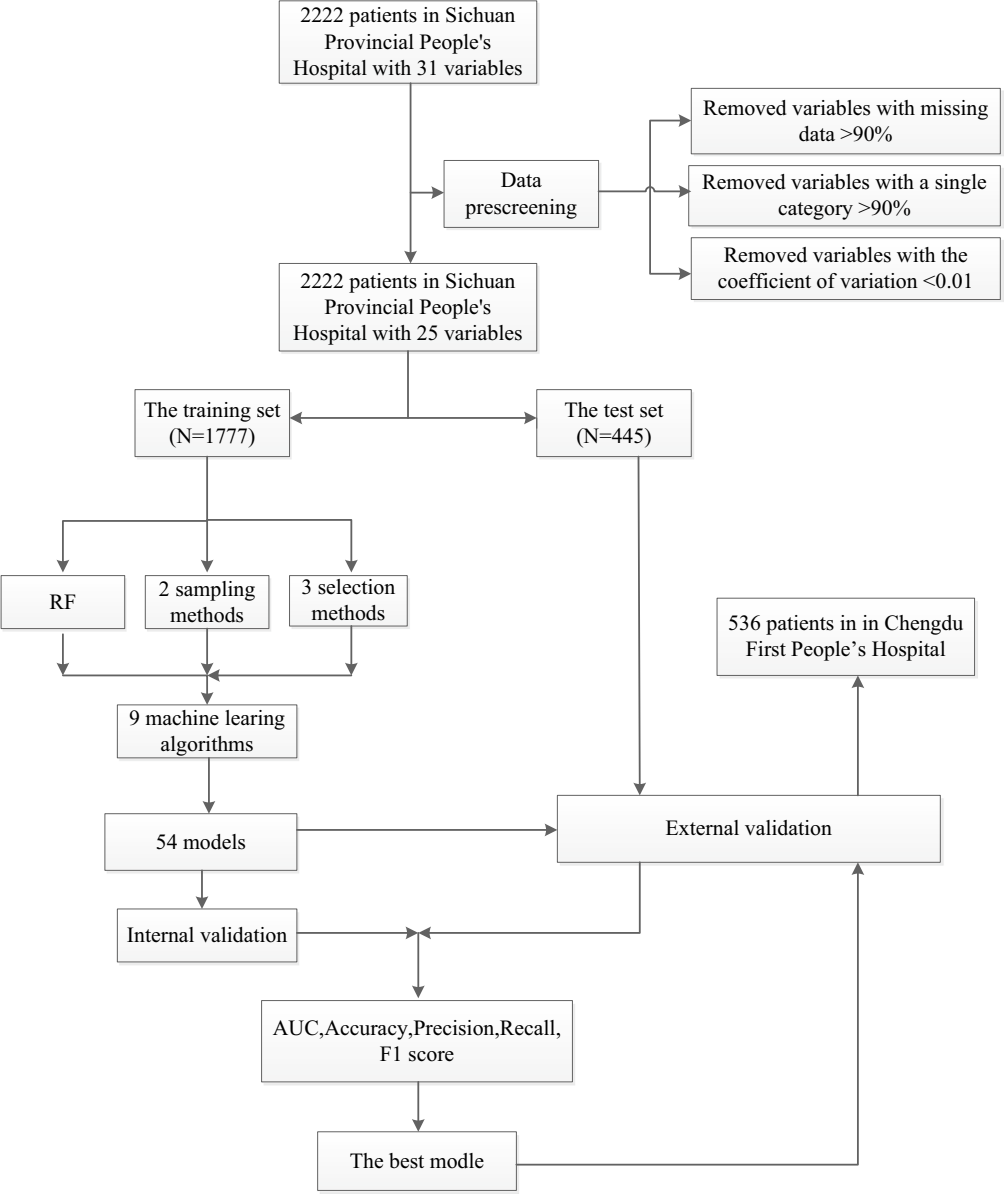
Flowchart. *RF* random forest, *AUC* area under the curve.

To minimize the adverse impact of data imbalance on prediction performance, the synthetic minority oversampling technique (SMOTE) and the borderline synthetic minority oversampling technique (BSMOTE) were applied. Three variable selection methods were used: (1) the Boruta screening method which is a feature selection algorithm to identify the minimal set of relevant variables; (2) the Lasso screening method which evaluates the importance of variables and output the results by introducing a penalization parameter penalizing and discarding unimportant variables; and (3) recursive feature elimination(RFE), which selects those features in a training dataset that are more or most relevant in predicting the target variable (Fig. 4)^31,32^.

### Model development

In this process, 9 machine learning algorithms were trained for binary classification and applied to develop predictive models, including logistic regression, random forest, stochastic gradient descent (SGD), extreme Gradient Boosting (XGBoost), K-nearest neighbor (KNN), support vector classify (SVC), decision tree, category boosting (CatBoost), multilayer perceptron (MLP)^31,32^. The dataset of Sichuan Provincial People's Hospital was divided into a training set and a test set at a ratio of 8:2; the training set was used to build models, and the test set was used to evaluate the predictive performance of the models. Internal validation was conducted with tenfold cross-validation in the training set (Fig. 4)^31^.

### Model evaluation

We used the AUC, accuracy, precision, recall rate, F1 value and area under the precision-recall curve (AUPRC) to evaluate the predictive performance of the model^31^. The AUCs of different models were compared, and the model with the largest AUC was selected to develop a PONV prediction system of PCA. SHAP helped to explain the contribution of variables to the model^31^. We applied the best model to patients in Chengdu First People’s Hospital and used the same quantitative metrics to evaluate the performance of the model (Fig. 4).

### Sample size validation

To estimate the impact of sample sizes on predictive performance, 10% of the samples were randomly extracted from the training set to train the model, and the AUC was evaluated in the test set. The training samples increased from 10 to 100% in increments of 10%. The above process was repeated 100 times, and the results were plotted on a line graph^31^. The contribution of a sample size to improve the prediction performance of models was assessed according to the inflection point change on the line graph.

### Statistical analysis

Continuous variables were described by mean and standard deviation, whereas categorical variables were expressed in terms of frequencies and percentages. Analysis of variance (ANOVA) and rank sum test were used for univariate analysis. Hypothesis testing and model building were implemented using the stats and sklearn packages in Python (V.3.8)^31^.

## Supplementary Information

Supplementary Table S1.

## Data Availability

All data generated or analyzed during this study are included in the paper and in the Supplementary Materials (Table S1). Raw data on this study are available from the corresponding author on reasonable request.
