# Supplementary material for: Development and assessment of novel machine learning models to predict the probability of postoperative nausea and vomiting for patient-controlled analgesia
Source: Sci Rep. 2023 Apr 20;13:6439. doi: 10.1038/s41598-023-33807-7 (PMC10119140; doi:10.1038/s41598-023-33807-7)
Supplement: Supplementary file 1 — Supplementary Table S1. [file 41598_2023_33807_MOESM1_ESM.docx]

**Table S1** Baseline characteristics of patients in Sichuan Provincial People's Hospital.

| **Variables** |  | **No PONV**  **(n=1913)** | **PONV**  **(n=309)** | ***P*-value** |
| --- | --- | --- | --- | --- |
| Type of surgery |  |  |  |  |
|  | Gastroenterology | 588(33.9%) | 100 (38.2%) |  |
|  | Thoracic surgery | 265(15.3%) | 33 (12.6%) |  |
|  | Hepatobiliary | 246(14.2%) | 55(21.0%) |  |
|  | Orthopedics | 435(25.1%) | 40(15.3%) |  |
|  | Urology | 203(11.5%) | 35(13.0%) |  |
|  | Gynecology | 176(9.20%) | 46(14.9%) |  |
| Age |  | 52.74±14.26 | 52.98±14.54 | 0.618 |
| Sex |  |  |  | <0.001 |
|  | Male | 859(44.9%) | 59(19.1%) |  |
|  | Female | 1054(55.1%) | 250(80.9%) |  |
| Height (cm) |  | 162.22± 7.96 | 158.73±7.40 | <0.001 |
| Weight (Kg) |  | 61.56 ±10.65 | 58.29± 9.61 | <0.001 |
| BMI |  | 23.51±3.54 | 23.07±3.21 | 0.307 |
| ASA grade |  |  |  | 0.313 |
|  | 1 | 224(11.7%) | 29(9.4%) |  |
|  | 2 | 1528(79.9%) | 255(82.5%) |  |
|  | 3 | 160(8.40%) | 25(8.1%) |  |
| Past medical history | No | 1327 (76.6%) | 238(77.0%) | 0.727 |
|  | Hypertension | 257(14.8%) | 42(13.6%) |  |
|  | Diabetes | 73 (4.2%) | 10(3.2%) |  |
|  | Both | 76 (4.4%) | 19(6.2%) |  |
| History of surgery |  |  |  | 0.460 |
|  | Without | 908 (48.0%) | 141(45.6%) |  |
|  | With | 1005 (52.0%) | 168(54.4%) |  |
| Smoking status |  |  |  | <0.001 |
|  | Without | 1594(83.3%) | 296(95.8%) |  |
|  | With | 319(16.7%) | 13(4.2%) |  |
| Alcohol Drinking |  |  |  | <0.001 |
|  | Without | 1692(88.4%) | 301(97.4%) |  |
|  | With | 221(11.6%) | 8(2.6%) |  |
| Laparoscopic surgery |  |  |  | 0.003 |
|  | Without | 640(33.5%) | 81(26.2%) |  |
|  | With | 1273(66.5%) | 228(73.8%) |  |
| Operative duration(h) |  |  |  | 0.049 |
|  | ≤1h | 16 (0.8%) | 4(1.2%) |  |
|  | 1~3h | 1018(53.2%) | 155 (50.2%) |  |
|  | 3~6h | 729(38.6%) | 118(38.2%) |  |
|  | >6h | 140(7.3%) | 32(10.4%) |  |
| Infusion volume(ml) |  | 1120.72±679.60 | 1228.71±707.95 | 0.004 |
| Intraoperative urine volume(ml) |  | 231.18±292.88 | 254.01±303.48 | 0.048 |
| Blood loss(ml) |  | 159.80±149.30 | 169.47±148.76 | 0.163 |
| Antiemetics in the surgery |  |  |  | 0.205 |
|  | No | 48(2.5%) | 9(2.9%) |  |
|  | Dexamethasone | 83(4.3%) | 12(3.9%) |  |
|  | Dexamethasone, Tropisetron | 1346(70.4%) | 208(67.3%) |  |
|  | Tropisetron | 436(22.8%) | 80(25.9%) |  |
| Non-opioids in the surgery |  |  |  | 0.057 |
|  | No | 147(7.7%) | 30(9.7%) |  |
|  | Dezocine | 1267(66.2%) | 211(68.3%) |  |
|  | Dezocine, Flurbiprofen | 390(20.4%) | 49(15.9%) |  |
|  | Flurbiprofen | 109(6.7%) | 19(6.1%) |  |
| Propofol |  |  |  |  |
|  | without | 0 | 0 |  |
|  | with | 1913(100%) | 309(100%) |  |
| Remifentanil consumption(mcg) |  | 975.19±539.07 | 1053.58±575.75 | 0.319 |
| Sufentanil consumption(mcg) |  | 29.42±14.68 | 29.61±9.16 | 0.981 |
| Midazolam |  |  |  | 0.501 |
|  | Without | 125(6.5%) | 22(7.1%) |  |
|  | With | 1788(93.5%) | 287(92.9%) |  |
| Ephedrine |  |  |  | 0.312 |
|  | Without | 1243(65.0%) | 197(63.8%) |  |
|  | With | 670(35.0%) | 112(36.2%) |  |
| Volatile anesthetics |  |  |  | 0.249 |
|  | Without | 140(7.25%) | 15(4.9%) |  |
|  | With | 1773(92.7%) | 294(95.1%) |  |
| Dexmedetomidine |  |  |  | 0.023 |
|  | Without | 406(21.2%) | 53(17.2%) |  |
|  | With | 1507(78.8%) | 256(82.8%) |  |
| Time of PACU (min) |  |  |  | 0.506 |
|  | No | 434(22.7%) | 75(21.3%) |  |
|  | ≤60 | 919(48.0%) | 137(44.3%) |  |
|  | >60 | 560(29.3%) | 97(31.4%) |  |
| Movement pain score in 24 h |  | 2.25±0.91 | 2.64±0.99 | <0.001 |
| Length of Stay(day) |  | 11.51±7.65 | 11.80±7.36 | 0.009 |
| Postoperative rescue analgesics |  |  |  | 0.579 |
|  | Without | 507(26.5%) | 91(29.4%) |  |
|  | With | 1406(73.5%) | 218(70.6%) |  |
| Antiemetics in the ward |  |  |  | <0.001 |
|  | Without | 1846(96.5%) | 270(87.4%) |  |
|  | With | 67 (3.5%) | 39(12.6%) |  |
| PCA regimen |  |  |  | 0.118 |
|  | Sufentanil | 1271(66.4%) | 216(69.9%) |  |
|  | Hydromorphone | 168(8.8%) | 28(9.1%) |  |
|  | Sufentanil, Non-steroidal anti-inflammatory drugs | 468(24.5%) | 63 (20.4%) |  |
|  | Others | 6(0.3%) | 2(0.6%) |  |
| History of motion sickness and/or PONV |  |  |  | <0.001 |
|  | No | 1178(61.6%) | 45(14.2%) |  |
|  | Yes | 735(38.4%) | 264 (85.8%) |  |

*PCA* patient-controlled analgesia, *PONV* postoperative nausea and vomiting, *ASA* American Society of Anesthesiologists, *PACU* postanesthesia care unit, *BMI* body mass index.
